# The 23-year tracking of blood lipids from adolescence to adulthood in Korea: the Kangwha study

**DOI:** 10.1186/s12944-017-0615-2

**Published:** 2017-11-22

**Authors:** Jung Hyun Lee, Hyeon Chang Kim, Dae Ryong Kang, Il Suh

**Affiliations:** 1 Department of Medicine, the Graduate School of Yonsei University, 50 Yonsei-ro, 03722, Seodaemun-gu, Seoul, South Korea; 2 Department of Preventive Medicine, Yonsei University College of Medicine, 50-1 Yonsei-ro, 03722, Seodaemun-gu, Seoul, South Korea; 3 Institute of Genomic Cohort, Yonsei University Wonju College of Medicine, 20 Ilsan-ro, 26426, Wonju, South Korea

**Keywords:** Lipids, Epidemiology, Risk factors, Adolescence, Tracking, Republic of Korea

## Abstract

**Background:**

Several studies have examined tracking pattern of blood lipids level during long follow-up periods in Western countries. However, there have been few such studies in Asian populations.

**Methods:**

The Kangwha Study is a community-based prospective cohort study that started in 1986 on Kangwha Island, South Korea. A total of 432 participants (47% men) were enrolled in the study, during which serum total cholesterol, triglycerides, and high-density lipoprotein (HDL) cholesterol levels were measured for each participant at least once during adolescence (12–16 years of age) and again at least once during adulthood (25–35 years of age). The tracking patterns of the blood lipid levels were determined using Spearman correlation coefficients and tracking coefficients from generalized estimating equations.

**Results:**

The Spearman correlation coefficients between lipid measurements ranged from 0.12 to 0.73 depending on the lipid profile and measurement time interval; all were significant (*p* < 0.05). The magnitude of the coefficients tended to decrease as the time interval increased. When adjusted for age, sex, body mass index, and blood pressure, the tracking coefficients were 0.58 (95% confidence interval [CI]: 0.54–0.63) for total cholesterol, 0.39 (95% CI: 0.31–0.48) for triglycerides, and 0.51 (95% CI: 0.47–0.56) for HDL cholesterol. In a subgroup analysis by sex, the tracking coefficients were higher for women than for men, except for HDL cholesterol.

**Conclusions:**

The tracking patterns of blood lipids from adolescence to adulthood were notable. This study supports the importance of measuring lipids during adolescence for identifying high-risk individuals.

**Electronic supplementary material:**

The online version of this article (10.1186/s12944-017-0615-2) contains supplementary material, which is available to authorized users.

## Background

Atherosclerotic cardiovascular disease (CVD) begins in the early periods of life, and childhood metabolic abnormalities may continue through later life [1–3]. Several longitudinal studies have demonstrated tracking patterns for CVD risk factors over a long-term period [4–7]. By definition, tracking patterns mean the correlation between early and later measurements over the lifetime with respect to a certain variable [8]. The existence of a tracking pattern for CVD risk factors indicates that the timely detection of CVD risk factors in early life may make it possible to predict adult CVD.

Dyslipidemia (an abnormal blood lipid profile level) is an established risk factor for CVD and premature death [9–11]. Several long-term studies have evaluated the tracking patterns of lipids in Western countries [3, 12, 13]. However, there is an ethnic disparity in the prevalence of dyslipidemia and lipid levels [14], and longitudinal data is lacking for Asian populations [15]. To our knowledge, a few longitudinal studies have evaluated the tracking patterns of lipids in Asian populations, but these studies had relatively short follow-up periods [16, 17].

Our study aims to evaluate the tracking patterns of blood lipid levels from adolescence to adulthood. Specifically, we wanted to demonstrate the tracking patterns for total cholesterol, triglycerides, high-density lipoprotein (HDL) cholesterol, non-HDL cholesterol, and low-density lipoprotein (LDL) cholesterol. We also assessed the magnitude of tracking coefficients for these lipids. Furthermore, the tracking patterns were evaluated according to sex and measurement time intervals.

## Methods

### Study participants

The Kangwha study is a community-based prospective cohort study that started in 1986 with 430 children on Kangwha Island, Korea [16, 18]. At the time, the participants in the Kangwha study were first-grade students in local elementary schools; most of them were 6 years old. Annual examinations were conducted during each participant’s childhood and adolescence (1987–1997). The number of participants was expanded several times to include other students who lived on Kangwha Island and were in the same grade as the participants during the same period. After these expansions, four follow-up studies were conducted when the participants were adults: adult wave 1 in 1999–2001, adult wave 2 in 2005, adult wave 3 in 2010–2011, and adult wave 4 in 2014–2016.

Lipid profiles were measured three times during adolescence: adolescence 1 in 1992, adolescence 2 in 1994, and adolescence 3 in 1996. A total of 896 participants were tested at least once during this period. In this study, we enrolled the 432 participants who had their lipid profiles measured at least once during adult waves 2–4. Each participant in our study had at least two (and up to six) lipid measurements. This study does not use the data from adult wave 1 because the participants were in a transition stage from adolescence to adulthood at that time. Informed consent was obtained from all participants in our study. The study protocol was approved by the institutional review board of Severance Hospital at Yonsei University Health System (4–2014-0914).

### Measurements

Standing height and weight were measured to 0.1 cm and 0.1 kg, respectively, for each study participant. Body mass index (BMI) was calculated by dividing weight (kg) by the square of the height (m^2^). Resting systolic blood pressure (SBP) and diastolic blood pressure (DBP) were measured from the right brachial artery by trained researchers. Lipid measurements were performed using blood samples after overnight fasting. Total cholesterol, triglycerides, and HDL cholesterol were measured using enzymatic methods: in adolescence, the Hitachi 747 (Hitachi, Japan); in adult wave 2, the Hitachi 7150 (Hitachi, Japan); in year 1 of adult wave 3, the ADVIA 1650 (Siemens, Princeton, NJ, USA); and in year 2 of adult wave 3 and adult wave 4, the ADVIA 1800 (Siemens, Princeton, NJ, USA). Non-HDL cholesterol was calculated by subtracting the HDL cholesterol level from the total cholesterol level. LDL cholesterol was calculated using the Friedewald formula when blood triglycerides were <4.52 mmol/L [19].

### Statistical analysis

The general characteristics of study participants are presented as means and standard deviations (SD) or numbers and percentages. Because not all study participants participated in all measurements, the number of participants may vary depending on the measurement year. To compare the differences in lipid levels and other characteristics between participants who were followed up to adulthood and those who withdrew from the study, Student’s *t*-test and the chi-square test were used.

The tracking patterns were evaluated using three methods. First, the tracking pattern was visualized using several figures that showed the changes in mean values of baseline quartile groups as age increased. For this purpose, each lipid profile value at 12 years of age (measurement year: 1992, *n* = 343) was divided into four groups according to the quartile value of each lipid level to obtain the maximum tracking time. The mean value of each baseline group at each follow-up examination is presented as a figure. Second, we used the Spearman correlation coefficient (ρ) for lipid profiles between each measurement year from adolescence to adulthood. The Spearman correlation coefficients are presented according to the measurement time intervals. Third, we used a tracking coefficient reported by Twisk [8] because the traditional Spearman correlation coefficients can only evaluate the correlation between two measurements. To calculate the tracking coefficients, we used a generalized estimating equation (GEE) to evaluate the overall correlation. The formula to calculate the tracking coefficient is as follows:

$$ {Y}_{it}={\beta}_0+{\beta}_1{Y}_{it1}+{\beta}_2t+\sum \limits_{j=1}^J{\beta}_{3j}{X}_{ijt}+{\varepsilon}_{it} $$

In this formula, *Y*_*it*_ is the observation for subject *i* at time *t*, *Y*_*it1*_ is the first observation for subject *i*, *X*_*ijt*_ is the *j*th covariate for subject *i* at time *t* (where the number of total covariates = *J*), and *ε*_*it*_ indicates the error term. Detailed information on the formula has been published elsewhere [8]. Among the regression coefficients *β*, the standardized *β*_*1*_ is the tracking coefficient. To obtain the standardized *β*_*1*,_ the SD of *Y*_*it1*_ was multiplied by *β*_*1*_, and then the SD of *Y*_*it*_ was divided by *β*_*1*._ The correlations between the initial measurements and all other remaining measurements were integrated into a single tracking coefficient, *β*. The tracking coefficient has competitive strength when unbalanced data sets are used because it can handle missing values and data with unequal time intervals. In addition, it allows for the adjustment of possible confounders, such as age and BMI.

To calculate the tracking coefficient for lipid profiles, we analyzed 1869 measurements from 432 participants. The tracking coefficients for men, women, and all participants were calculated. We analyzed the adjusted tracking coefficient by adjusting for sex, age, BMI, and SBP at each measurement. To solve the problem of multiple comparisons, the Bonferroni correction was used. The study participants took part in up to six measurements, so *p* values of <0.008 were considered to be statistically significant for this comparison. Otherwise, unless noted, *p* values of <0.05 were considered to be statistically significant. All analyses were performed using SAS version 9.4 (SAS Institute Inc., Cary, NC, USA).

## Results

Table 1 shows the general characteristics of the study participants at each measurement. The mean ages of participants were 12 years, 14 years, 16 years, 25 years, 30 years, and 35 years of age at adolescence 1–3 and adult waves 2–4, respectively. The measurement periods were longer during adult waves 3 and 4 than in previous waves; therefore, the SDs for the participants’ age in adult waves 3 and 4 are larger than those of participants before adult wave 3. The total cholesterol levels decreased during adolescence and increased in adulthood. Non-HDL cholesterol and LDL cholesterol levels showed similar patterns. BMI, SBP, and DBP tended to increase as age increased.

**Table 1:** General characteristics of study participants for six measurement periods

Characteristics	Adolescence 1	Adolescence 2	Adolescence 3	Adult wave 2	Adult wave 3	Adult wave 4
*n*	342	346	402	275	262	247
Age, years	12.96 ± 0.29	14.97 ± 0.29	16.96 ± 0.30	25.75 ± 0.32	30.73 ± 0.57	35.91 ± 0.66
Sex (% men)	163 (47.7)	168 (48.6)	188 (46.8)	126 (45.8)	123 (47.0)	136 (55.1)
Total cholesterol, mmol/L	4.19 ± 0.66	4.10 ± 0.70	3.93 ± 0.69	4.24 ± 0.72	4.76 ± 0.87	4.97 ± 0.85
Triglyceride, mmol/L	1.18 ± 0.47	1.21 ± 0.51	1.24 ± 0.68	1.02 ± 0.64	1.17 ± 0.88	1.48 ± 0.99
HDL cholesterol, mmol/L	1.23 ± 0.22	1.15 ± 0.22	1.16 ± 0.25	1.49 ± 0.31	1.30 ± 0.32	1.45 ± 0.36
Non-HDL cholesterol, mmol/L	2.96 ± 0.61	2.95 ± 0.64	2.78 ± 0.65	2.75 ± 0.69	3.46 ± 0.83	3.52 ± 0.89
LDL cholesterol, mmol/L^a^	2.42 ± 0.57	2.39 ± 0.59	2.21 ± 0.62	2.28 ± 0.61	2.93 ± 0.66	2.85 ± 0.77
Body mass index, kg/m^2b^	18.90 ± 3.08	20.18 ± 2.98	21.03 ± 2.67	21.82 ± 3.01	23.09 ± 3.54	23.65 ± 3.44
Systolic blood pressure, mmHg^b^	111.57 ± 10.56	116.62 ± 11.53	116.08 ± 11.68	118.60 ± 14.35	116.13 ± 14.22	116.99 ± 13.83
Diastolic blood pressure, mmHg^b^	65.42 ± 9.39	67.62 ± 9.49	66.56 ± 8.53	69.90 ± 8.43	70.06 ± 9.17	75.70 ± 10.22

Abbreviations: *HDL* high-density lipoprotein, *LDL* low-density lipoprotein

Values are expressed as means ± standard deviations or numbers (%). ^a^The number of participants was somewhat different due to missing values (400 at adolescence 3, 258 at adult wave 3, and 244 at adult wave 4). ^b^The number of participants was somewhat different due to missing values (340 at adolescence 1, 345 at adolescence 2, and 400 at adolescence 3)

**Fig. 1:**
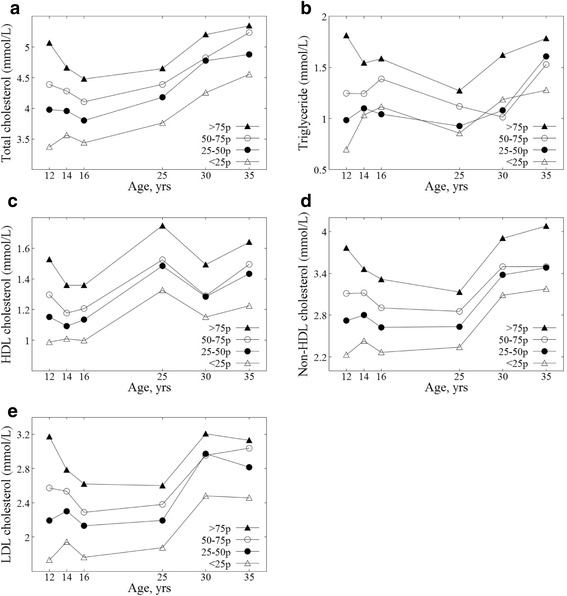
Tracking patterns of lipid levels for study participants from adolescence to adulthood**. a** Total cholesterol. **b** Triglycerides. **c** HDL cholesterol. **d** Non-HDL cholesterol. **e** LDL cholesterol. Each point in the figures represents the mean value of lipid levels in the baseline quartile group (at 12 years of age, *n* = 342) at each measurement: at 14 years of age, *n* = 335; at 16 years of age, *n* = 313; at 25 years of age, *n* = 222; at 30 years of age, *n* = 199; at 35 years of age, *n* = 186 for total cholesterol. (The number of participants at each measurement for LDL cholesterol was somewhat different due to missing values)

Figure 1 shows the tracking patterns for the lipid levels of study participants from ages 12 to 35 years according to the mean values of the quartile groups at 12 years of age (adolescence 1, *n* = 342). The >75p group includes the participants who had higher lipid levels than the 75th percentile value at 12 years of age. The 50–75p group includes the participants who had lipid levels between the 50th and 75th percentile values by age. The 25–50p group includes participants who had lipid levels between the 25th and 50th percentile values by age. The <25 group includes participants who had lower lipid levels than the 25th percentile values by age. In the absence of a tracking pattern, the mean values for each quartile group would gradually converge. The trends for total cholesterol, HDL cholesterol, non-HDL cholesterol, and LDL cholesterol showed similar sustained differences among each quartile groups, while the difference of mean values between the 25th–50th and the 50th–75th percentile groups decreased at age 30 years, as shown in Fig. 1. Triglycerides showed an irregular tracking pattern.

To evaluate the statistical significance of tracking patterns, Spearman correlation coefficients were calculated (Table 2). All coefficients were significant based on *p* values (all *p* values were <0.05); however, the magnitude of these values shows a difference. The range of coefficients was 0.38–0.69 for total cholesterol, 0.12–0.66 for triglycerides, and 0.41–0.69 for HDL cholesterol. The coefficients for non-HDL cholesterol and LDL cholesterol showed a similar trend as those of total cholesterol and HDL cholesterol. In general, as the time interval increased, the magnitude of coefficients tended to decrease.

**Table 2:** Spearman correlation coefficients between the lipid levels of study participants according to measurement intervals

Correlation of lipid measurements by different intervals	n	Total cholesterol	Triglycerides	HDL cholesterol	Non-HDL cholesterol	LDL cholesterol^a^
23-year correlation (age 12 to 35 years)	186	0.41	0.17	0.43	0.34	0.37
21-year correlation (age 14 to 35 years)	186	0.44	0.27	0.46	0.39	0.35
19-year correlation (age 16 to 35 years)	230	0.46	0.32	0.49	0.42	0.43
18-year correlation (age 12 to 30 years)	199	0.38	0.12	0.41	0.36	0.38
16-year correlation (age 14 to 30 years)	203	0.49	0.33	0.58	0.44	0.45
14-year correlation (age 16 to 30 years)	251	0.48	0.32	0.51	0.44	0.44
13-year correlation (age 12 to 25 years)	222	0.46	0.21	0.45	0.44	0.44
11-year correlation (age 12 to 35 years)	225	0.53	0.28	0.52	0.45	0.45
10-year correlation (age 25 to 35 years)	126	0.61	0.66	0.64	0.69	0.62
9-year correlation (age 16 to 25 years)	259	0.51	0.37	0.54	0.45	0.46
5-year correlation (age 25 to 30 years)	154	0.69	0.57	0.69	0.69	0.64
5-year correlation (age 30 to 35 years)	167	0.63	0.56	0.66	0.68	0.63
4-year correlation (age 12 to 16 years)	313	0.59	0.34	0.58	0.64	0.52
2-year correlation (age 12 to 14 years)	335	0.62	0.39	0.62	0.65	0.58
2-year correlation (age 14 to 16 years)	322	0.69	0.45	0.63	0.73	0.66

Abbreviations: *HDL* high-density lipoprotein, *LDL* low-density lipoprotein

All *p* values of correlation coefficients were <0.05. ^a^The number of participants for calculating the correlation coefficients of LDL cholesterol was somewhat different due to missing values

Table 3 shows the tracking coefficients, which were 0.58 (95% confidence interval [CI]: 0.54–0.63) for total cholesterol, 0.39 (0.31–0.48) for triglycerides, and 0.51 (0.47–0.56) for HDL cholesterol when adjusted for age, sex, BMI, and SBP at each measurement. The tracking coefficients of non-HDL cholesterol and LDL cholesterol (which were calculated from other lipid measurements, such as total cholesterol, triglycerides, and HDL cholesterol) were not higher than those of total cholesterol. The tracking coefficients were consistent in their magnitude before and after adjustment. In a subgroup analysis by sex, the tracking coefficients were higher in women than in men, except for HDL cholesterol. The tracking coefficient of HDL cholesterol was the highest compared to other coefficients for men, whereas the tracking coefficient of total cholesterol was the highest for women.

**Table 3:** Unadjusted and adjusted tracking coefficients of lipid levels for study participants from adolescence to adulthood according to sex

Lipids	Number of participants	Average number of measurements per person	Tracking coefficient (95% CI)	
			Unadjusted	Adjusted^a^
Total				
Total cholesterol	432	4.33	0.58 (0.54–0.62)	0.58 (0.54–0.63)
Triglyceride	432	4.33	0.40 (0.32–0.49)	0.39 (0.31–0.48)
HDL cholesterol	432	4.33	0.54 (0.49–0.60)	0.51 (0.47–0.56)
Non-HDL cholesterol	432	4.33	0.56 (0.52–0.61)	0.56 (0.52–0.60)
LDL cholesterol	432	4.30	0.57 (0.52–0.61)	0.56 (0.52–0.61)
Men				
Total cholesterol	205	4.40	0.52 (0.46–0.58)	0.52 (0.46–0.58)
Triglyceride	205	4.40	0.44 (0.31–0.56)	0.39 (0.26–0.52)
HDL cholesterol	205	4.40	0.58 (0.52–0.64)	0.56 (0.51–0.62)
Non-HDL cholesterol	205	4.40	0.51 (0.45–0.58)	0.50 (0.44–0.56)
LDL cholesterol	205	4.34	0.50 (0.43–0.57)	0.50 (0.43–0.57)
Women				
Total cholesterol	227	4.26	0.68 (0.62–0.73)	0.67 (0.61–0.72)
Triglyceride	227	4.26	0.42 (0.36–0.49)	0.41 (0.35–0.48)
HDL cholesterol	227	4.26	0.52 (0.45–0.59)	0.50 (0.43–0.56)
Non-HDL cholesterol	227	4.26	0.69 (0.63–0.74)	0.65 (0.59–0.71)
LDL cholesterol	227	4.26	0.65 (0.59–0.71)	0.63 (0.57–0.69)

Abbreviations: *HDL* high-density lipoprotein, *CI* confidence interval

All *p* values of tracking coefficients were <0.008. ^a^Adjusted for sex, measurement year, body mass index, and systolic blood pressure at each measurement

## Discussion

In this long-term follow-up study of Korean adolescents, blood lipid levels showed notable tracking patterns. When compared to the tracking patterns of other lipids, the tracking pattern of triglycerides was the weakest. Because there is no criterion standard for evaluating tracking patterns of multiple measurements [8], we used several methods in our study. Traditionally, Pearson and Spearman correlation coefficients have been used for evaluating the tracking patterns between two measurements. This study used Spearman correlation coefficients when we were assessing the correlation between lipid measurements, because lipids were not normally distributed in our data. The interpretation of Spearman correlation coefficients between two measurements is relatively simple; negligible, 0.0–0.3; low positive, 0.3–0.5; moderate positive, 0.5–0.7; high positive, 0.7–0.9; and very high positive, 0.9–1.0. [20]; however, it becomes difficult when multiple measurements are involved. Therefore, we calculated the tracking coefficients using the GEE method [4, 8] to find an integrated value for multiple lipid measurements for tracking.

We considered the unequal time intervals between measurements, age, sex, SBP, and BMI to be possible confounders. We chose BMI and SBP as possible confounders to evaluate the independent tracking pattern of lipids because the tracking of obesity was reported to be stronger than for other CVD risk factors [15]; in addition, a previous study using the Kangwha Study data showed a noticeable tracking pattern of SBP from childhood to adulthood [18].

Our results showed consistency in the magnitude of tracking coefficients before and after the adjustment was made. This indicates that the effect of confounders on lipid tracking is not large. Both genetic and environmental factors can affect blood lipids tracking from adolescence to adulthood, although it is unclear which factors are more influential. One twin study in China shows the tracking patterns for total cholesterol, triglyceride, and LDL cholesterol were predominantly influenced by genetic factor [21]. Our results might be affected by the genetically determined level of each individual. However, further longitudinal study which evaluates the influence on lipid tracking of both genetic and environmental factors is needed as a future work.

The results from the Spearman correlation coefficients (Table 2) and the tracking coefficients using the GEE method (Table 3) are consistent in our study. When evaluating the tracking phenomenon of repeatedly measured values over a long-term period, some values ​​measured at short intervals can inflate the tracking coefficients of the entire period. Therefore, time-specific Spearman correlation coefficients and unified tracking coefficients are required to adequately evaluate long-term tracking coefficients. Figure 1 shows the overall tracking patterns of lipid levels for study participants from adolescence to adulthood, as well as similar tracking patterns to Tables 2 and 3.

A few studies have addressed the tracking of blood lipid changes from adolescence to adulthood. The Busselton study [12] showed that the correlation coefficients of cholesterol for tracking ranged from 0.35 to 0.55; in addition, the coefficient for a shorter time interval between measurements tended to show a strong correlation among adolescents at baseline. The Pune Children’s Study [7] reported 13-year correlation coefficients with a distribution of 0.26–0.53 from childhood to early adulthood. Our results are consistent with these previous studies, although our participants had different ages and were from a different country. One previous study [22] reported somewhat different results for lipid tracking with respect to sex and ethnicity, but it did not include Asian adolescents and only had a short follow-up period. A few studies tracked lipids in children and adolescents in East Asia [16, 17]. However, no study has been performed in East Asia to track lipids from adolescence to adulthood. Thus, our results should contribute to the understanding of long-term lipid tracking patterns in an East Asian population.

We mainly used tracking coefficients that were calculated using GEE to determine tracking patterns. We categorized the coefficients as follows, based on a previous study: low, ≤0.30; moderate, 0.30–0.59; moderately high, 0.60–0.89; and high, ≥0.9 [5]. Our study shows that significant correlation exists among lipid profile measurements, with tracking coefficients in the 0.39–0.67 range (Table 3). For women, the tracking coefficients for total cholesterol, non-HDL cholesterol, and LDL cholesterol showed moderately high correlation; other tracking coefficients showed moderate correlation according to the above criteria. Several studies have used tracking coefficients to determine tracking patterns. Ulmer et al. [5] reported tracking coefficients for blood lipids in the range of 0.62–0.66 for men and 0.63–0.69 for women over 15 years. Wilsgaard et al. [4] showed tracking coefficients for blood lipids in the range of 0.43–0.77 for men and 0.39–0.64 for women over 16 years. Twisk et al. [23] reported tracking coefficients for serum HDL cholesterol of 0.51 for men and 0.65 for women during adulthood. Our results for tracking coefficients were similar with these previously reported results.

In our study, the tracking coefficients for triglycerides were generally lower than those for other lipid profiles. The low coefficients for triglycerides might be affected by inappropriate fasting in the study population during adolescence examinations. In addition, women showed higher tracking coefficients than men did in our study, except for HDL cholesterol. Considering that sexual maturity varies by age, the difference in lipid tracking patterns with respect to sex might be due to the effects of sex hormones during adolescence [24, 25]. The roles of non-HDL and LDL cholesterol have been emphasized in the prediction of CVD, especially for children and adolescents [15, 26]. We calculated non-HDL and LDL cholesterol and evaluated their tracking patterns; however, there was no noticeable increase compared to those of total and HDL cholesterols. Further studies are needed to evaluate the usefulness of non-HDL and LDL cholesterol levels at adolescent for predicting CVD.

Lipid screening during adolescence is controversial [15, 27]. In 2011, the US National Heart, Lung, and Blood Institute recommended that universal lipid screening tests be conducted twice in adolescence and young adulthood, at 9–11 years and 17–21 years of age [15]. In 2016, the US Preventive Services Task Force concluded that there was insufficient evidence to recommend lipid screenings for children and adolescents [27]. It is difficult to apply such guidelines directly to Asian countries because there are disparities in lipid levels among different ethnicities [14]. Moreover, long-term follow-up periods have been lacking in studies of lipid tracking in Asian populations. Thus, further epidemiological research is necessary to determine the benefits of lipid screenings in early periods of life for Asian populations. Although recent studies showed that adolescent CVD risk factors can predict the future risk of developing clinical CVD, the usefulness of mass screening at adolescence may vary depending on the magnitude of long-term tracking of risk factor levels [28, 29]. In this regard, our study demonstrated notable tracking patterns for lipids in Koreans from adolescence to adulthood, which supports the importance of the early measurement of lipids.

Our study had several strengths. First, to the best of our knowledge, this study is the first to investigate the tracking patterns of lipid profiles from adolescence to adulthood in East Asia. The long follow-up period—more than 23 years—is also a strong point of this study. Second, the study participants had similar ages and residences at adolescence; thus, the potential confounding effects of age or residence should be minimized. Third, we used various methods to evaluate tracking patterns and derived an integrated tracking coefficient. Using these methods, we were able to account for missing values, reflect unequal time intervals, and adjust for other potential confounders, such as BMI and SBP.

However, there are some limitations to this research. First, our study participants do not represent the Korean population as a whole. Thus, we compared our study participants to the individuals of same age from the Korea National Health and Nutrition Examination Survey. In the study, the mean values of total cholesterol level of men were 4.11 mmol/L for 12–13 years of age, 3.88 for 14–15, and 3.83 for 16–17, while the mean values of women were higher than those of men; 4.16 mmol/L for 12–13 years of age, 4.13 for 14–15, and 4.19 for 16–17, respectively [30]. These lipids levels and trends do not show large difference compared to our study results. But, the results of our study should be applied carefully to other populations. Second, our study had a low follow-up rate. To overcome this limitation, we conducted comparison analyses for general characteristics at adolescence 1 between participants who were followed up to adulthood and those who withdrew before adulthood. Most characteristics did not show significant differences between the groups (See Additional file 1: Table S1). Third, the lack of lipid profile measurements in childhood was also another limitation. Further research which considers the entire human lifespan is needed. Fourth, the change of devices for lipid measurement is also a limitation. It can cause measurement error. There are several studies regarding reliability of lipid measurement, but results are controversial [31, 32]. Fifth, we could not analyze genetic or behavioral risk factors which can affect lipids levels and tracking patterns due to lack of related data. Finally, we could not obtain reliable information on any medication that might affect the tracking patterns of our participants. Generally, lipid-lowering drugs would stabilize the lipid levels of participants who had abnormal lipid levels. In this regard, our results might be less affected.

## Conclusions

Our study showed a moderately high tracking pattern for blood lipids from adolescence to adulthood. Triglycerides showed slightly irregular patterns in some age groups, although other cholesterols (e.g., total, HDL, non-HDL, LDL) showed consistent patterns in all age groups. As the time interval increased, the correlations for lipids decreased; however, all coefficient correlations were significant. The tracking coefficients were greater for women than for men, except in the case of HDL cholesterol. Our results support the usefulness of early lipid measurements for identifying high-risk individuals in advance based on the tracking patterns of lipids.

## Additional file

Additional file 1: Table S1. Comparison of baseline characteristics (at 12 years) between participants who were followed up to adulthood and those who withdrew before adulthood. (DOCX 28 kb)

## Abbreviations

BMI: Body mass index
CI: Confidence interval
CVD: Cardiovascular disease
DBP: Diastolic blood pressure
GEE: Generalized estimating eq.
HDL: High-density lipoprotein
LDL: Low-density lipoprotein
SBP: Systolic blood pressure
SD: Standard deviation
