# Supplementary material for: The 23-year tracking of blood lipids from adolescence to adulthood in Korea: the Kangwha study
Source: Lipids Health Dis. 2017 Nov 22;16:221. doi: 10.1186/s12944-017-0615-2 (PMC5700514; doi:10.1186/s12944-017-0615-2)
Supplement: Additional file 1: Table S1. — Comparison of baseline characteristics (at 12 years) between participants who were followed up to adulthood and those who withdrew before adulthood. (DOCX 28 kb) [file 12944_2017_615_MOESM1_ESM.docx]

**Supplementary Table 1. Comparison of baseline characteristics (at 12 years) between participants who were followed up to adulthood and those who withdrew before adulthood**

| **Characteristics** | **Participants who were followed up to adulthood** | | **Participants who withdrew before adulthood** | | ***p* value** |
| --- | --- | --- | --- | --- | --- |
|  | **Number** | **Mean (SD)** | **Number** | **Mean (SD)** |  |
| Age, years | 342 | 12.96 ± 0.29 | 357 | 13.01 ± 0.32 | 0.035 |
| Sex (% men) | 163 | (47.7) | 169 | (46.3) | 0.775 |
| Total cholesterol, mmol/L | 342 | 4.19 ± 0.66 | 365 | 4.21 ± 0.69 | 0.691 |
| Triglyceride, mmol/L | 342 | 1.18 ± 0.47 | 365 | 1.24 ± 0.74 | 0.184 |
| HDL cholesterol, mmol/L | 342 | 1.23 ± 0.22 | 365 | 1.22 ± 0.27 | 0.609 |
| Non-HDL cholesterol, mmol/L | 342 | 2.96 ± 0.61 | 365 | 2.99 ± 0.63 | 0.522 |
| LDL cholesterol, mmol/L | 342 | 2.42 ± 0.57 | 364 | 2.43 ± 0.59 | 0.809 |
| Body mass index, kg/m^2^ | 340 | 18.90 ± 3.08 | 363 | 18.97 ± 3.22 | 0.748 |
| Systolic blood pressure, mmHg | 340 | 111.57 ± 10.56 | 364 | 111.28 ± 10.27 | 0.712 |
| Diastolic blood pressure, mmHg | 340 | 65.42 ± 9.39 | 364 | 65.15 ± 9.06 | 0.692 |
| Abbreviations: HDL, high-density lipoprotein; LDL, low-density lipoprotein.  Data are expressed as means ± standard deviations or number (%). The number of participants was somewhat different due to missing values. | | | | | |
